# Supplementary material for: Relationship between anion gap and 28-day all-cause mortality in patients with acute pulmonary edema: A retrospective analysis of the MIMIC-IV database
Source: PLoS One. 2025 Sep 26;20(9):e0333293. doi: 10.1371/journal.pone.0333293 (PMC12468886; doi:10.1371/journal.pone.0333293)
Supplement: Attachment — Submitted filename: Response to Reviewers.docx [file pone.0333293.s003.docx]

**Journal Requirements:**

When submitting your revision, we need you to address these additional requirements.

**1. Please ensure that your manuscript meets PLOS ONE's style requirements, including those for file naming. The PLOS ONE style templates can be found at**

**https://journals.plos.org/plosone/s/file?id=wjVg/PLOSOne_formatting_sample_main_body.pdf and**

[**https://journals.plos.org/plosone/s/file?id=ba62/PLOSOne_formatting_sample_title_authors_affiliations.pdf**](https://journals.plos.org/plosone/s/file?id=ba62/PLOSOne_formatting_sample_title_authors_affiliations.pdf)

**Response:**

Thank you for your careful review and valuable comments on our manuscript. We have thoroughly revised our manuscript to ensure that it meets all the style requirements of PLOS ONE, including those for file naming. Here are the specific actions we have taken:

1. File Naming: We have renamed the manuscript file to clearly reflect the title of our study and the revision status, as per the journal's guidelines.

2. Manuscript Format: We have reviewed and adjusted the manuscript to comply with PLOS ONE's formatting guidelines. This includes:

2.1 Ensuring the title page includes the full title, authors' names, affiliations, and contact information.

2.2 Formatting the abstract to be concise and informative, summarizing the key aspects of our study.

2.3 Structuring the manuscript into clear sections: Introduction, Methods, Results, and Discussion.

2.4 Ensuring all figures and tables are clearly labeled and referenced within the text.

2.5 Formatting the references according to PLOS ONE's citation style.

3. Content Review: We have also reviewed the content to ensure clarity, coherence, and adherence to the journal's standards for scientific reporting.

Thank you for your understanding and support. We look forward to your positive response.

**2. We note that your Data Availability Statement is currently as follows:**

**“All relevant data are within the manuscript and its Supporting Information files”**

**Please confirm at this time whether or not your submission contains all raw data required to replicate the results of your study. Authors must share the “minimal data set” for their submission. PLOS defines the minimal data set to consist of the data required to replicate all study findings reported in the article, as well as related metadata and methods (https://journals.plos.org/plosone/s/data-availability#loc-minimal-data-set-definition).**

**For example, authors should submit the following data:**

**- The values behind the means, standard deviations and other measures reported;**

**- The values used to build graphs;**

**- The points extracted from images for analysis.**

**Authors do not need to submit their entire data set if only a portion of the data was used in the reported study.**

**If your submission does not contain these data, please either upload them as Supporting Information files or deposit them to a stable, public repository and provide us with the relevant URLs, DOIs, or accession numbers. For a list of recommended repositories, please see https://journals.plos.org/plosone/s/recommended-repositories.**

**If there are ethical or legal restrictions on sharing a de-identified data set, please explain them in detail (e.g., data contain potentially sensitive information, data are owned by a third-party organization, etc.) and who has imposed them (e.g., an ethics committee). Please also provide contact information for a data access committee, ethics committee, or other institutional body to which data requests may be sent. If data are owned by a third party, please indicate how others may request data access.**

**Response:**

Thank you for your prompt response and for bringing this matter to our attention. We confirm that our submission contains all the raw data required to replicate the results of our study. In accordance with PLOS ONE's data availability policy, we have included the “minimal data set” necessary to replicate all study findings reported in the article, as well as related metadata and methods.

If there are any further requirements or if you need additional information, please let us know. We are committed to ensuring full compliance with PLOS ONE's data availability policy.

Thank you for your understanding and support.

**3. Please review your reference list to ensure that it is complete and correct. If you have cited papers that have been retracted, please include the rationale for doing so in the manuscript text, or remove these references and replace them with relevant current references. Any changes to the reference list should be mentioned in the rebuttal letter that accompanies your revised manuscript. If you need to cite a retracted article, indicate the article’s retracted status in the References list and also include a citation and full reference for the retraction notice.**

**Response:**

Thank you for your attention to the reference list in our manuscript. We have carefully reviewed our reference list to ensure its completeness and accuracy. We have also checked for any retracted papers and have made the necessary adjustments.

During our review, we found that all the references in our list are relevant, current, and have not been retracted. Therefore, no changes were needed in this regard. We have ensured that all citations are up-to-date and accurately reflect the current state of the literature.

We have double-checked the reference list to ensure that it is formatted correctly and that all necessary information is included for each citation. This includes the authors' names, article titles, journal names, volume numbers, page ranges, and publication years.

We have also reviewed the text of the manuscript to ensure that all references are properly cited and that the citations are consistent with the information provided in the reference list.

Thank you for your understanding and support. We look forward to your positive response.

**Reviewer #1:** This is a well-conducted and technically sound retrospective cohort study exploring the prognostic role of anion gap levels in patients with acute pulmonary edema using the MIMIC-IV database. The methodology is appropriate and clearly described, the statistical analyses are rigorous (including multivariable Cox regression, restricted cubic spline, Kaplan-Meier survival analysis, and subgroup interaction testing), and the conclusions are well-supported by the presented data.

Strengths of the study include:

Use of a large, publicly available and high-quality ICU dataset (MIMIC-IV).

Clear stratification of patients into quartiles of anion gap and appropriate adjustments for confounders.

Subgroup analysis exploring interactions with age and renal disease, which adds valuable clinical insight.

**Suggestions for Improvement:**

**Q1. Language and Grammar: While the manuscript is overall intelligible and written in acceptable English, several minor grammatical and typographical issues persist. A language polishing service or editorial revision may enhance clarity and flow.**

**Response:**

Thank you for your constructive feedback regarding the language and grammar of our manuscript. We have carefully reviewed the manuscript and made the following revisions to enhance clarity and flow:

1. Abstract:

We have replaced "used" with "utilized" to make the expression more formal.

We have changed "divided" to "categorized" to more accurately reflect the classification of patients.

We have added "indicating that" to clarify the logical connection in the subgroup analysis sentence.

2. Introduction:

We have simplified the sentence structure by removing "the accumulation of" to make it more concise.

We have replaced "although" with "while" to improve the fluency of the sentence.

We have changed "due to" to "given" to make the sentence more formal.

3. Methods:

We have replaced "covers" with "encompasses" to more accurately describe the scope of the MIMIC-IV database.

We have changed "were" to "included" to make the sentence more formal.

4. Results:

We have replaced "with" with "and" to improve the coherence of the sentence.

We have changed "revealed" to "demonstrated" to make the expression more formal.

We have replaced "showing" with "demonstrating" to make the sentence more formal.

5. Discussion:

We have replaced "consistent with" with "in line with" to make the expression more formal.

We have changed "aligns with" to "is consistent with" to improve the formality of the sentence.

We have replaced "multiple" with "several" to make the sentence more concise.

6. Limitations:

We have replaced "susceptible to" with "prone to" to make the expression more formal.

We have changed "introduces" to "may introduce" to make the sentence more cautious.

We have replaced "did not further explore" with "did not delve into" to make the expression more formal.

7. Conclusion:

We have replaced "demonstrates" with "indicates" to make the expression more formal.

We have changed "could be integrated" to "could be incorporated" to make the sentence more formal.

We believe these revisions have improved the overall clarity and flow of the manuscript. We have also conducted a thorough language check to ensure that no other grammatical or typographical issues remain. We appreciate your understanding and look forward to your positive response.

**Q2. Figures and Tables: Ensure that all figures (e.g., Kaplan-Meier curves, RCS curves, forest plots) are of high resolution and clearly labeled for publication quality.**

**Response:**

Thank you very much for your attention to and suggestions on our manuscript. We have carefully addressed the revision issue regarding the figures and tables, and here is our detailed response:

1. Enhancement of Image Resolution and Clarity

We have redrafted and optimized all figures, including the Kaplan-Meier curves (Figure 3), restricted cubic spline curves (Figure 2), and forest plots (Figure 4). High-resolution drawing tools were utilized, and the resolution of each image was set to no less than 300dpi to meet the high-quality requirements for publication clarity. As a result, the details and lines of the images are now presented more clearly, enabling readers to observe and comprehend the data visualization and analysis outcomes more accurately.

2. Clarification and Standardization of Image Labels

To ensure the readability and comprehensibility of the images, we have thoroughly reviewed and optimized the labels of all figures. We have ensured that titles, axis labels, legends, and other elements in each image are clear, accurate, and legible. For the Kaplan-Meier curves, we have distinctly labeled the survival curves of different groups and clearly differentiated them in the legend. In the restricted cubic spline curves, we have meticulously labeled the names and units of the independent and dependent variables and added necessary annotations at appropriate positions in the figure to facilitate readers' understanding of the nonlinear relationship depicted. For the forest plots, we have standardized the labels of each subgroup, making them more concise and clear, and clearly displayed the effect sizes and confidence intervals of different subgroups in the figure.

3. Consistency Check Between Images and Text Content

We have also carefully checked the consistency between the image content and the text description. We have ensured that the data and analysis results presented in the images are completely consistent with the content mentioned in the text, avoiding any potential misunderstandings or confusions. Through this consistency check, we have further improved the overall quality and accuracy of the manuscript, enabling the images and text to complement and support each other better in conveying clear and accurate research information.

We believe that with these improvements, our figures and tables have now met the high-quality standards for publication. We look forward to your further feedback and are willing to continue making necessary adjustments and optimizations according to your suggestions to ensure that the manuscript is presented in the best possible form to readers.

Thank you again for your patient guidance and assistance!

**Q3. Ethics and Data Availability: While the MIMIC-IV database is exempt from IRB due to its anonymized nature, it may still benefit clarity to explicitly cite the IRB approval from PhysioNet and mention the CITI certification more formally within the Methods.**

**Response:**

Thank you for your valuable comments. We have carefully revised the manuscript according to your suggestions. Here is the reply to the specific revision request:

We have revised the “Data Source” section in the Methods to explicitly state the IRB approval from PhysioNet and more formally mention the CITI certification. The revised text now reads:

“The data were sourced from the MIMIC-IV database, which covers patient data from 2008 to 2019. The MIMIC-IV database is an anonymized dataset, and its use for research purposes is exempt from Institutional Review Board (IRB) approval due to its anonymized nature. However, to ensure ethical compliance, the study obtained approval from the PhysioNet IRB. Author Guang Tu completed the CITI Data or Specimens Only Research course, obtained approval for database access, and assumed responsibility for data extraction (certification number 65828445).”

Additionally, we have updated the “Compliance with Ethics Guidelines” section to further clarify the ethical approval process:

“Compliance with Ethics Guidelines: The research adhered to the principles outlined in the 1964 Helsinki Declaration and subsequent revisions. Approval for this endeavor was granted by the review boards of both the Massachusetts Institute of Technology (MIT) and Beth Israel Deaconess Medical Center (BIDMC), with an exemption from obtaining informed consent. Additionally, the study obtained approval from the PhysioNet IRB to use the anonymized MIMIC-IV dataset for research purposes.”

We believe these changes enhance the clarity and transparency of our ethical and data handling procedures.

**Q4. Limitations: The limitations are well addressed. However, a brief mention of potential misclassification bias in coding diagnoses (from EHR-based databases) would further strengthen the discussion.**

**Response:**

Thank you for your constructive feedback. We have carefully considered your suggestion regarding the potential misclassification bias in coding diagnoses from EHR-based databases and have incorporated this into our discussion of limitations.

We have added a specific mention of the potential for misclassification bias in coding diagnoses within the “Limitations” section of our manuscript. This addition highlights the potential inaccuracies in the classification of acute pulmonary edema and other comorbidities due to the use of an Electronic Health Record (EHR)-based database. The revised text now reads:

“Our study has several limitations. First, as a retrospective study, it is prone to selection bias and the influence of confounding factors. Although we adjusted for multiple potential confounders through multivariate analysis, there may still be unidentified or unadjusted confounding factors. Additionally, the specific diagnostic criteria for acute pulmonary edema were not uniformly documented in the MIMIC-IV database, which may introduce potential variability in the diagnosis of acute pulmonary edema among patients. This limitation is inherent to the retrospective nature of the study and the reliance on existing medical records. Second, our study relied solely on data from the MIMIC-IV database, which may have issues with data completeness and accuracy. Additionally, we were unable to obtain detailed clinical interventions for the patients, which could affect the assessment of prognosis. Third, there is a potential for misclassification bias in coding diagnoses, as the data are derived from an Electronic Health Record (EHR)-based database. This could lead to inaccuracies in the classification of acute pulmonary edema and other comorbidities. Lastly, our study did not delve into the specific mechanisms underlying elevated anion gap levels, and future research is needed to validate these mechanisms and explore targeted treatment strategies based on anion gap monitoring. Moreover, since our study was limited to patients in the MIMIC-IV database, the results may not be fully generalizable to patient populations in other regions or different healthcare settings. Therefore, future studies should be conducted in broader patient populations to validate our findings and further explore their clinical application value.”

We believe that this addition strengthens our discussion by acknowledging another potential source of bias and by providing a more comprehensive view of the limitations inherent in our study design.

Thank you again for your valuable input.

**Q5. Clinical Implications: Consider slightly expanding the clinical implications in the conclusion—for example, how the anion gap could be integrated into early risk stratification or ICU scoring systems.**

**Response:**

Thank you for your suggestion regarding the clinical implications of our study. We have revised the conclusion section of the discussion to better highlight the potential clinical applications of our findings.

We have expanded the clinical implications section in the conclusion to emphasize the potential integration of the anion gap into early risk stratification tools and ICU scoring systems. The revised text now reads:

“Our study demonstrates that admission anion gap levels predict 28-day all-cause mortality in patients with acute pulmonary edema, particularly in younger patients and those without renal disease. Clinically, the anion gap could be integrated into early risk stratification tools or ICU scoring systems to help identify high-risk patients more promptly. This could enhance the precision of prognostic assessments and guide clinical decision-making. Further research is warranted to elucidate the underlying mechanisms and to develop targeted therapeutic strategies based on anion gap monitoring.”

Additionally, we have added a sentence in the preceding paragraph to further elaborate on the clinical relevance:

“Therefore, in clinical practice, it is essential to consider these factors comprehensively to more accurately assess patient conditions and outcomes. The anion gap, as an easily measurable parameter, could potentially be incorporated into early risk stratification protocols or used to refine existing ICU scoring systems, thereby enhancing the precision of prognostic assessments.”

We believe these revisions provide a clearer picture of the potential clinical applications of our findings and how they could be integrated into clinical practice.

Thank you again for your valuable feedback.

**Reviewer #2:** I have read this manuscript and I haver some questions.

**Q1.** **In the introduction, the authors do not tell me why this topic is important. Many conditions cause an anion gap. why should we be concerned about an elevated anion gap in a patient with pulmonary edema. It is not until the discussion that the authors tell us that prior studies have shown an association and that the association may be a marker of significant systemic inflammatory response. This information should be in the introduction.**

**Response:**

Thank you very much for your insightful comments. We fully agree with your suggestion that the importance of the topic and the rationale for focusing on the anion gap in patients with pulmonary edema should be highlighted in the Introduction section. We have revised the manuscript accordingly to address this issue.

Specifically, we have added the following paragraph after the sentence “Early identification and intervention are crucial to improve outcomes, yet the clinical presentation can be variable and challenging to assess accurately.” in the Introduction section:

“Notably, while many conditions can lead to an elevated anion gap, it may hold particular significance in patients with acute pulmonary edema. Prior studies have demonstrated an association between elevated anion gap levels and adverse outcomes in critically ill patients, and this association may serve as a marker of a significant systemic inflammatory response. Thus, exploring the relationship between anion gap levels and prognosis in patients with acute pulmonary edema is of great importance for better understanding the severity of the condition and guiding therapeutic decisions.”

We believe that this revision effectively addresses your concern by clearly stating the importance of the topic and the potential significance of an elevated anion gap in patients with pulmonary edema at the beginning of the manuscript. This change enhances the logical flow and coherence of the article, allowing readers to understand the importance and necessity of the study from the outset.

Thank you again for your valuable feedback. We have also made several other minor revisions to improve the clarity and readability of the manuscript. The revised manuscript is attached for your review.

**Q2. The authors stated that the patients were diagnosed with acute pulmonary edema. What was the diagnostic criteria and was is the same for all of the patients. I understand that this is a retrospective cohort so there may not be a way to get this information. If this is the case, this should be stated in the discussion.**

**Response:**

Thank you for raising this important point regarding the diagnostic criteria for acute pulmonary edema in our study. We appreciate your attention to this detail.

In response to your query, we have carefully reviewed our methodology and have made appropriate revisions to both the Methods and Discussion sections of our manuscript to address the potential variability in diagnostic criteria.

In the Methods section, we have added a statement acknowledging that the specific diagnostic criteria for acute pulmonary edema were not uniformly documented in the MIMIC-IV database. This reflects the inherent variability in clinical practice and is a limitation of our retrospective study design. We have included this information to ensure transparency about the potential impact on our findings.

Additionally, in the Discussion section, we have expanded on this limitation, emphasizing that the lack of uniform diagnostic criteria introduces potential variability in the diagnosis of acute pulmonary edema among patients. We have highlighted that this is a common challenge in retrospective cohort studies that rely on existing medical records, and we have discussed how this limitation might affect the interpretation of our results.

We believe these revisions provide a more comprehensive understanding of the study's methodology and the potential biases associated with the diagnostic criteria for acute pulmonary edema. We have also ensured that these limitations are clearly communicated to the readers.

Thank you again for your valuable feedback. We have attached the revised manuscript for your review.

**Q3. Lastly, the authors use the MIMIC-IV database. I would like for the authors to describe this database. Why did they choose this database. What is the purpose of this data base. Where do the patients come from?**

**Response:**

Thank you for your inquiry regarding the MIMIC-IV database. We appreciate the opportunity to provide further details about this valuable resource and our rationale for using it in our study.

1. Description of the MIMIC-IV Database

The MIMIC-IV (Medical Information Mart for Intensive Care IV) database is a large, publicly available database that contains comprehensive clinical data from patients admitted to the intensive care units (ICUs) of Beth Israel Deaconess Medical Center in Boston, Massachusetts, USA. The database covers a wide range of information, including demographics, vital signs, laboratory results, procedures, medications, and more. It is designed to support research in critical care medicine by providing detailed and high-quality data from a large cohort of patients.

2. Reasons for Choosing the MIMIC-IV Database

2.1 Comprehensive Data: The MIMIC-IV database offers a rich and detailed dataset that includes a wide array of clinical variables, which is essential for conducting thorough and robust research. This allows us to explore various factors and their associations with outcomes in patients with acute pulmonary edema.

2.2 Large Sample Size: The database contains data from a large number of patients, which increases the statistical power of our study and enhances the generalizability of our findings.

2.3 High-Quality Data: The data in MIMIC-IV are meticulously collected and curated, ensuring high accuracy and reliability. This is crucial for generating valid and meaningful results.

2.4 Public Availability: The MIMIC-IV database is freely accessible to researchers, which promotes transparency and reproducibility in research. This aligns with our commitment to rigorous and open scientific inquiry.

3. Purpose of the Database

The primary purpose of the MIMIC-IV database is to serve as a resource for research in critical care medicine. It aims to facilitate the study of various aspects of critical illness, including pathophysiology, treatment efficacy, and patient outcomes. By providing detailed and high-quality data, the database supports the development of new insights and improvements in the care of critically ill patients.

4. Patient Population

The patients included in the MIMIC-IV database are those admitted to the ICUs of Beth Israel Deaconess Medical Center. The data cover a diverse patient population, reflecting a wide range of clinical conditions and demographics. This diversity is important for ensuring that the findings from studies using the database are applicable to a broad range of clinical settings and patient groups.

5. Conclusion

In summary, the MIMIC-IV database is an invaluable resource for critical care research due to its comprehensive data, large sample size, high quality, and public availability. These features make it an ideal choice for our study on the relationship between anion gap levels and mortality in patients with acute pulmonary edema. We believe that using this database allows us to conduct a rigorous and impactful investigation that can contribute valuable insights to the field.

Thank you again for your thoughtful review and feedback.
