# Supplementary material for: Relationship between anion gap and 28-day all-cause mortality in patients with acute pulmonary edema: A retrospective analysis of the MIMIC-IV database
Source: PLoS One. 2025 Sep 26;20(9):e0333293. doi: 10.1371/journal.pone.0333293 (PMC12468886; doi:10.1371/journal.pone.0333293)
Supplement: Attachment — Submitted filename: Response_to_Reviewers_auresp_2.docx [file pone.0333293.s004.docx]

**Journal Requirements:**

**1. If the reviewer comments include a recommendation to cite specific previously published works, please review and evaluate these publications to determine whether they are relevant and should be cited. There is no requirement to cite these works unless the editor has indicated otherwise.**

**Response:**

Thank you for your valuable feedback. We have carefully reviewed the comments provided by the reviewers and would like to address the specific point regarding the citation of previously published works.

We understand the importance of citing relevant literature to support our findings and to situate our work within the existing body of knowledge. However, the reviewers did not provide specific recommendations to cite particular previously published works. Therefore, we have focused on ensuring that our references are comprehensive and relevant to the study's context and findings.

We have double-checked our references to confirm that they accurately reflect the current state of research in our field and are pertinent to our study's objectives, methods, and conclusions. We believe that our current list of citations adequately covers the necessary literature and provides a solid foundation for our work.

We appreciate the opportunity to clarify this matter and are confident that our manuscript is now fully aligned with the expectations for academic rigor and scholarly contribution.

Thank you for your continued guidance and support.

**2. Please review your reference list to ensure that it is complete and correct. If you have cited papers that have been retracted, please include the rationale for doing so in the manuscript text, or remove these references and replace them with relevant current references. Any changes to the reference list should be mentioned in the rebuttal letter that accompanies your revised manuscript. If you need to cite a retracted article, indicate the article’s retracted status in the References list and also include a citation and full reference for the retraction notice.**

**Response:**

Thank you for your attention to detail and for highlighting the importance of maintaining an accurate and up-to-date reference list in our manuscript. We have conducted a thorough review of our references to ensure completeness and correctness.

We have checked each citation against the most recent databases and have verified that none of the papers we have cited have been retracted. We understand the significance of retractions in the academic record and have taken the necessary steps to ensure that our references reflect the current state of the literature.

In the event that a retracted paper is deemed essential for the context of our study, we would follow the guidelines you provided. We would include a rationale for citing the retracted work within the manuscript text, clearly indicating its retracted status in the References list. Additionally, we would provide a citation and full reference for the retraction notice to maintain transparency and to acknowledge the current status of the cited work.

We have made no changes to the reference list in this revision, but we will adhere to these practices should the need arise in the future. We believe that our reference list is now complete and correct, and it supports the scholarly integrity of our manuscript.

We appreciate your guidance and are committed to upholding the highest standards of academic research and publication ethics.

Thank you for your continued support.

**Reviewer #3:** General comments

This study addresses an important and clinically relevant question by evaluating the prognostic value of a simple, routinely available biomarker the anion gap in patients with acute pulmonary edema. The use of robust statistical methods, including Cox regression, restricted cubic splines (RCS), and Kaplan Meier analysis, strengthens the methodological rigor. The conclusion is practical and clearly linked to the study findings, offering suggestions that could translate into better bedside risk stratification.

**Specific comments**

**Methods section**

**Q1. Patient identification: It would improve clarity to explain exactly how patients with acute pulmonary edema were identified in the database such as through ICD codes, clinician-entered diagnoses, or the first charted diagnosis.**

**Response:**

Thank you very much for your insightful comments and suggestions. We highly appreciate your efforts in reviewing our manuscript and are pleased to address your concerns.

We have revised the relevant section of the manuscript to provide a more detailed explanation of how patients with acute pulmonary edema were identified in the MIMIC-IV database. Specifically, we identified patients with acute pulmonary edema using the ICD codes J810 and 5184. These codes were used to extract the initial cohort of patients who were clinically diagnosed with acute pulmonary edema at admission.

We hope these revisions address your concerns. Thank you again for your valuable feedback and for giving us the opportunity to improve our manuscript.

**Q2. Timing: Please clarify whether the anion gap value used was the very first measurement within the first 24 hours of admission or an average across several measurements.**

**Response:**

Thank you for your valuable feedback on our manuscript. We are pleased to address your question regarding the timing of the anion gap measurement.

We have clarified that the anion gap value used in our analysis was the first measurement within the first 24 hours of admission. This approach was chosen to ensure that we captured the initial metabolic state of the patients upon admission, which is crucial for assessing the potential impact of acute conditions on anion gap levels. We believe that using the first measurement provides a more accurate reflection of the patients' baseline condition at the time of admission.

We hope this clarification addresses your concern. Thank you once again for your insightful comments, which have helped us improve the clarity of our manuscript.

**Q3. Missing data: It would help readers to know how missing values were handled whether patients with missing key variables were excluded, or if imputation methods were applied.**

**Response:**

Thank you very much for your valuable comments and suggestions. We have carefully addressed the issue regarding the handling of missing data in our revised manuscript. Here is our detailed response:

In our revised manuscript, we have clearly described how missing data were handled. Specifically:

1. Exclusion of Patients with Missing Key Variables:

(1) Patients with missing anion gap data were excluded from the study. This decision was made because the anion gap is a critical variable in our analysis, and its absence would compromise the accuracy and reliability of our results.

(2) For other variables, if the proportion of missing data exceeded 50%, the corresponding patients were also excluded. This criterion ensures that our dataset remains robust and suitable for analysis.

2. Multiple Imputation for Variables with Less Than 50% Missing Data:

(1) For variables with less than 50% missing data, we applied multiple imputation by chained equations (MICE). This method generates multiple complete datasets to handle missing values, thereby reducing bias and improving the integrity of our analysis. The specific steps are as follows:

• Using the MICE algorithm to impute missing values and generate multiple complete datasets.

- Analyzing each complete dataset separately.
- Combining the results from multiple datasets to obtain the final statistical findings.

Specific Description

In the “Data collection” section, we explicitly stated:

“Patients with missing anion gap data were excluded. For other variables with missing values, if the missing proportion was greater than 50%, the corresponding patients were excluded. For other variables with missing values less than 50%, multiple imputation by chained equations was applied.”

We believe that these methods effectively reduce the bias caused by missing data and enhance the reliability of our study results. We hope this response clarifies how we handled missing data in our study.

Thank you once again for your careful review and feedback. We appreciate your time and effort in helping us improve our manuscript.

**Q4. Covariates: Consider listing all variables included in the multivariate Cox regression to show transparency and reproducibility.**

**Response:**

Thank you very much for your insightful comments and suggestions regarding the transparency and reproducibility of our study. We appreciate your attention to detail and the opportunity to enhance the clarity of our manuscript.

In response to your suggestion to list all variables included in the multivariate Cox regression analysis, we have made revisions to the manuscript. Specifically, we have added a detailed description of the covariates included in the multivariate Cox regression models in the Methods section under the “Statistical analysis” paragraph. The covariates included in the models are age, gender, race, myocardial infarction, heart failure, cerebrovascular disease, chronic pulmonary disease, diabetes, renal disease, SAPS II score, and SOFA score.

Additionally, we have ensured that the footnote of Table 3 accurately reflects the adjustments made in each model. This footnote now explicitly lists all the covariates included in the multivariate analysis, ensuring consistency with the Methods section and enhancing the transparency of our study.

We believe that these revisions will provide readers with a clear understanding of the variables considered in our analysis, thereby improving the reproducibility of our study. We are grateful for your guidance and hope that these changes meet your expectations.

Thank you once again for your valuable feedback.

**Q5. Sample size: Even in retrospective analyses, a note on whether a sample size calculation or justification for the final sample size was done would strengthen the methodological robustness.**

**Response:**

We appreciate your suggestion regarding the need for a sample size calculation or justification in our retrospective analysis. You are correct that even in retrospective studies, providing a rationale for the sample size can enhance the methodological robustness of the study.

In our study, we utilized data from the MIMIC-IV database, which is a large, comprehensive, and anonymized dataset containing detailed clinical information from 2008 to 2019. The primary objective of our study was to investigate the association between admission anion gap levels and 28-day all-cause mortality in patients with acute pulmonary edema. Given the nature of the MIMIC-IV database and the retrospective design of our study, the sample size was determined based on the availability of data that met our inclusion and exclusion criteria.

Specifically, we included adult patients diagnosed with acute pulmonary edema who had anion gap measurements within the first 24 hours of hospitalization. The final sample size of 1094 patients was determined by the number of eligible patients in the MIMIC-IV database who met these criteria. While we did not perform a formal sample size calculation prior to the analysis, we believe that the large sample size derived from the MIMIC-IV database provides sufficient statistical power to detect significant associations and draw meaningful conclusions.

To further address your concern, we have added a note in the Methods section of our manuscript to clarify this point:

“No formal sample size calculation was performed for this retrospective analysis. The final sample size was determined based on the availability of data in the MIMIC-IV database that met the inclusion and exclusion criteria.”

We hope this clarification addresses your concern regarding the sample size in our study. We have also revised the manuscript accordingly to reflect this explanation.

Thank you again for your careful review and valuable feedback. We believe that these revisions have improved the quality and clarity of our manuscript.

**Q6. Grouping: Explain why quartiles were chosen instead of using clinically meaningful cut-offs that might reflect metabolic acidosis severity.**

**Response:**

Thank you very much for your insightful comments regarding the grouping method in our study. We understand the suggestion to use clinically meaningful cut-offs for grouping; however, we chose to use quartiles for the following reasons:

1. Uniform Distribution of Data: Quartile grouping ensures an even distribution of patients across groups, which helps to avoid statistical biases that may arise from uneven sample sizes. This method allows us to comprehensively cover the entire range of anion gap levels and analyze their relationship with mortality.

2. Exploration of Non-linear Relationships: By using quartiles, we can more clearly observe the non-linear relationship between anion gap levels and mortality. This approach helps to reveal the impact of anion gap on prognosis at different levels, rather than focusing solely on fixed clinical thresholds.

3. Alignment with Study Objectives: Our primary objective was to investigate the relationship between anion gap levels and 28-day all-cause mortality in patients with acute pulmonary edema, rather than directly assessing the clinical severity of metabolic acidosis. Therefore, quartile grouping aligns better with our study design and objectives.

While we acknowledge that using clinically meaningful cut-offs might directly reflect the severity of metabolic acidosis in some contexts, we believe that quartile grouping is more appropriate for our study given the reasons mentioned above. Additionally, we have discussed the clinical significance of anion gap levels in the discussion section to help readers better understand their application in clinical practice.

Thank you again for your valuable feedback. We hope these explanations clarify our rationale for using quartile grouping.

**Q7. RCS: Specify the number and placement of knots used in the restricted cubic spline analysis, or mention if default settings were applied.**

**Response:**

We thank you for raising this important point about the restricted cubic spline (RCS) analysis. In our study, we used the default settings provided by the statistical software for the number and placement of knots in the RCS analysis. This approach is commonly used in similar studies to ensure a balanced and data-driven distribution of knots, which helps in capturing the non-linear relationships effectively.

To address your request for more transparency, we have added a specific note in the Methods section of our manuscript to clarify this:

“In the restricted cubic spline analysis, the default settings of the statistical software were applied for the number and placement of knots. This approach ensures a balanced distribution of knots based on the data, which is particularly useful for capturing non-linear relationships.”

We believe that using the default settings provided by the statistical software is a standard and reliable method for conducting RCS analysis. These settings are designed to optimize the placement of knots based on the data distribution, thereby providing a robust and unbiased assessment of the relationship between anion gap levels and 28-day all-cause mortality.

Thank you again for your valuable feedback. We hope this clarification addresses your concern regarding the RCS analysis.

**Q8. Subgroup analyses: It would add clarity to list all subgroups examined and explain briefly how interaction terms were tested.**

**Response:**

Thank you for your valuable feedback on our manuscript. We have taken your suggestion to enhance the clarity of our subgroup analysis by listing all subgroups examined and explaining how interaction terms were tested. Below are the modifications we have made in response to your comments:

Modifications in the "Methods" Section

Original Text:

"Subgroup analyses were conducted to explore the stability of the relationship between anion gap levels and mortality in different subgroups, including age and renal disease status."

Modified Text:

"We conducted subgroup analyses to explore the stability of the relationship between anion gap levels and mortality across various clinical subgroups. These subgroups included age (<65 years vs. ≥65 years), gender (male vs. female), race (white, black, other), history of myocardial infarct (yes vs. no), history of heart failure (yes vs. no), cerebrovascular disease (yes vs. no), chronic pulmonary disease (yes vs. no), diabetes (yes vs. no), and renal disease (yes vs. no). Interaction terms were tested using Cox regression models to assess the significance of the interaction between anion gap levels and each subgroup variable on 28-day all-cause mortality. The interaction terms were calculated as the product of the anion gap variable and each subgroup indicator variable. A p-value less than 0.05 was considered statistically significant for the interaction terms."

We believe these changes will provide the necessary clarity and detail regarding our subgroup analysis as suggested. We appreciate your guidance and are confident that these revisions will strengthen our manuscript.

Thank you once again for your constructive feedback.

**Q9. References: Consider adding references for the MIMIC-IV database and the statistical software packages used to help readers trace the data source and analytical tools.**

**Response:**

Thank you for your suggestion to enhance the transparency and reproducibility of our study by adding references for the MIMIC-IV database and the statistical software packages used. We have incorporated these references into our manuscript as follows:

Original Text:

"All statistical analyses were conducted utilizing R Statistical Software (Version 4.2.2, available at http://www.R-project.org, The R Foundation) and the Free Statistics Analysis Platform (Version 2.1, developed in Beijing, China, accessible via http://www.clinicalscientists.cn/freestatistics)."

Modified Text:

"All statistical analyses were conducted utilizing R Statistical Software (Version 4.2.2, available at http://www.R-project.org, The R Foundation) and the Free Statistics Analysis Platform (Version 2.1, developed in Beijing, China, accessible via http://www.clinicalscientists.cn/freestatistics). Data were sourced from the MIMIC-IV database, which encompasses patient data from 2008 to 2019[9]. The MIMIC-IV database is an anonymized dataset, and its use for research purposes is exempt from Institutional Review Board (IRB) approval due to its anonymized nature."

We believe these changes will help readers trace the data source and analytical tools more effectively. We appreciate your guidance and are confident that these revisions will further strengthen our manuscript.

Thank you once again for your constructive feedback.

**Discussion**

**Q10. It would strengthen the discussion to mention whether the association between anion gap and mortality remained after adjusting for comorbidities or disease severity scores like SOFA or SAPS II, if available.**

**Response:**

Thank you for suggesting that we discuss whether the association between anion gap and mortality remains significant after adjusting for comorbidities or disease severity scores like SOFA or SAPS II. We have incorporated this important point into our discussion section to provide a more comprehensive analysis of our findings.

In our study, we performed multivariate Cox regression analyses to assess the relationship between anion gap levels and 28-day all-cause mortality. We adjusted for a wide range of potential confounders, including age, gender, race, comorbidities (such as myocardial infarction, heart failure, cerebrovascular disease, chronic pulmonary disease, diabetes, and renal disease), and disease severity scores (SOFA and SAPS II). The results showed that the association between anion gap levels and mortality remained significant even after these adjustments (HR = 1.11, 95% CI: 1.07-1.15, *P* < 0.001). This indicates that anion gap levels provide additional prognostic value beyond traditional severity scores and comorbidities.

We have added the following text to the discussion section to highlight this important finding:

"In our study, the association between anion gap levels and mortality remained significant even after adjusting for comorbidities and disease severity scores such as SOFA and SAPS II. This suggests that anion gap levels provide additional prognostic value beyond traditional severity scores and comorbidities."

We believe that this addition strengthens the discussion by providing a more nuanced understanding of the independent prognostic significance of anion gap in patients with acute pulmonary edema. We appreciate your suggestion and believe it has improved the manuscript.

Thank you once again for your valuable feedback and for taking the time to review our manuscript.

**Q11. Highlight how this study adds new evidence or differs from previous studies on anion gap and ICU mortality (e.g., by focusing specifically on acute pulmonary edema patients).**

**Response:**

Thank you for suggesting that we highlight how our study adds new evidence or differs from previous studies on anion gap and ICU mortality. We have incorporated this important point into our discussion section to provide a clearer understanding of the unique contributions of our research.

Our study specifically focuses on patients with acute pulmonary edema, a specific patient population that has not been extensively explored in previous research on anion gap and mortality. While prior studies have demonstrated a strong correlation between elevated anion gap levels and adverse outcomes in critically ill patients, our study provides new evidence by demonstrating that anion gap levels are independently associated with 28-day mortality in patients with acute pulmonary edema. This finding highlights the importance of monitoring anion gap levels in this specific patient group and underscores the potential clinical value of anion gap as a prognostic marker in acute pulmonary edema.

We have added the following text to the discussion section to highlight this important point:

"Our study specifically focuses on patients with acute pulmonary edema, adding new evidence to the literature by demonstrating that anion gap levels are independently associated with 28-day mortality in this specific patient population. This finding highlights the importance of monitoring anion gap levels in patients with acute pulmonary edema and underscores the potential clinical value of anion gap as a prognostic marker in this context. Previous studies have shown associations between anion gap and mortality in various critical illnesses, but our study is one of the few that specifically examines this relationship in acute pulmonary edema patients."

We believe that this addition strengthens the discussion by clearly delineating the unique contributions of our study and how it differs from previous research. We appreciate your suggestion and believe it has improved the manuscript.

Thank you once again for your valuable feedback and for taking the time to review our manuscript.

**Q12. The suggestion of incorporating anion gap into early warning or risk scoring systems is valuable; consider naming widely used scores like APACHE II or SOFA and discussing what incremental value the anion gap might add.**

**Response:**

Thank you for your suggestion regarding the incorporation of anion gap into early warning or risk scoring systems. We agree that this is a valuable point and have expanded our discussion to include specific references to widely used scoring systems such as APACHE II and SOFA, as well as the potential incremental value that anion gap might add.

In our revised discussion, we have highlighted that the anion gap, as an easily measurable parameter, could potentially be incorporated into early risk stratification protocols or used to refine existing ICU scoring systems such as APACHE II or SOFA. The incremental value of anion gap in these scoring systems may lie in its ability to reflect metabolic disturbances and systemic inflammatory responses more sensitively. By integrating anion gap levels into these scores, clinicians may be able to identify high-risk patients more accurately and implement timely interventions to improve outcomes.

We have added the following text to the discussion section to address this point:

"The anion gap, as an easily measurable parameter, could potentially be incorporated into early risk stratification protocols or used to refine existing ICU scoring systems such as APACHE II or SOFA. The incremental value of anion gap in these scoring systems may lie in its ability to reflect metabolic disturbances and systemic inflammatory responses more sensitively. By integrating anion gap levels into these scores, clinicians may be able to identify high-risk patients more accurately and implement timely interventions to improve outcomes."

We believe that this addition strengthens the discussion by providing a more detailed and practical consideration of how anion gap could be utilized in clinical settings to enhance prognostic assessments. We appreciate your suggestion and believe it has improved the manuscript.

Thank you once again for your valuable feedback and for taking the time to review our manuscript.

**Q13. You might also discuss whether repeated measurement of anion gap over time (i.e., dynamic monitoring) could provide more prognostic information than a single admission value.**

**Response:**

Thank you very much for your valuable comments and suggestions. We have carefully considered your feedback and have revised our manuscript accordingly. In response to your suggestion regarding the potential benefits of dynamic monitoring of the anion gap over time, we have added the following discussion in our revised manuscript:

Response to Reviewer’s Comment:

We appreciate your insightful suggestion regarding the potential benefits of dynamic monitoring of the anion gap over time. While our study focused on the admission anion gap levels as a predictor of 28-day all-cause mortality in patients with acute pulmonary edema, we agree that repeated measurements of the anion gap could provide more comprehensive prognostic information. Dynamic monitoring of the anion gap could potentially capture changes in metabolic status and systemic inflammatory responses more sensitively than a single admission value. This approach may help clinicians track the progression of metabolic disturbances and adjust therapeutic strategies accordingly, potentially improving patient outcomes.

In our study, we identified that elevated anion gap levels at admission are independently associated with higher mortality, particularly in younger patients and those without renal disease. However, the anion gap can fluctuate during hospitalization due to various factors, including changes in tissue perfusion, inflammatory responses, and renal function. By monitoring the anion gap dynamically, clinicians could identify early signs of worsening metabolic acidosis or systemic inflammation, which might indicate a deteriorating clinical condition. This information could be particularly valuable in guiding interventions such as fluid management, vasopressor use, or renal replacement therapy.

Future research should explore the feasibility and clinical utility of dynamic anion gap monitoring in patients with acute pulmonary edema. Longitudinal studies that track anion gap levels over time could provide more detailed insights into its prognostic value and help refine clinical decision-making processes. Additionally, investigating the correlation between changes in anion gap levels and specific clinical outcomes could further enhance our understanding of its role in acute pulmonary edema management.

Once again, we thank you for your constructive feedback, which has significantly improved the quality of our manuscript.

**Limitations**

**Q14. It would add depth to acknowledge residual confounding from unmeasured factors like lactate or albumin, which directly affect the anion gap.**

**Response:**

Thank you very much for your insightful comments and suggestions. We greatly appreciate your feedback, which has helped us improve the quality of our manuscript.

In response to your suggestion regarding the acknowledgment of residual confounding from unmeasured factors such as lactate or albumin, which directly affect the anion gap, we have made the following revisions:

1. In the Discussion section:

We have added a statement to acknowledge the potential for residual confounding from unmeasured factors such as lactate or albumin. Specifically, we have included the following text in the Discussion section:

"However, it is important to acknowledge that residual confounding from unmeasured factors such as lactate or albumin, which directly affect the anion gap, may still exist."

2. In the Limitations section:

We have also added a similar acknowledgment in the Limitations section to highlight this potential source of bias:

"Lastly, our study did not delve into the specific mechanisms underlying elevated anion gap levels, and future research is needed to validate these mechanisms and explore targeted treatment strategies based on anion gap monitoring. Additionally, investigating the potential benefits of dynamic monitoring of anion gap levels over time, rather than relying on a single admission value, could provide more comprehensive prognostic information. Dynamic monitoring may help clinicians track changes in metabolic status and adjust therapeutic strategies accordingly, potentially improving patient outcomes. Moreover, since our study was limited to patients in the MIMIC-IV database, the results may not be fully generalizable to patient populations in other regions or different healthcare settings. Future prospective studies or multicenter validation studies are proposed to confirm these findings and assess the generalizability of our results to broader patient populations. It is also important to consider the potential for residual confounding from unmeasured factors such as lactate or albumin, which directly affect the anion gap."

We believe that these revisions add depth to our discussion and provide a more comprehensive acknowledgment of the limitations of our study. We appreciate your suggestion and have incorporated it to enhance the clarity and thoroughness of our manuscript.

Thank you once again for your valuable feedback.

**Q15. Consider adding a forward-looking statement proposing future prospective studies or multicenter validation to confirm these findings and assess generalizability.**

**Response:**

Thank you very much for your valuable suggestion regarding the addition of a forward-looking statement proposing future prospective studies or multicenter validation to confirm our findings and assess generalizability.

We have incorporated your suggestion into our manuscript as follows:

1. In the Discussion section:

We have added a forward-looking statement at the end of the Discussion section to emphasize the importance of future research. The added text reads:

“Future prospective studies or multicenter validation studies are proposed to confirm these findings and assess the generalizability of our results to broader patient populations.”

This statement highlights our recommendation for future research to validate our findings through prospective studies or multicenter validation, ensuring that our results are applicable to a wider range of patients.

2. In the Limitations section:

Similarly, we have included a forward-looking statement at the end of the Limitations section to reiterate the need for future research:

“Future prospective studies or multicenter validation studies are proposed to confirm these findings and assess the generalizability of our results to broader patient populations.”

This reiteration underscores the importance of conducting future studies in different regions and healthcare settings to validate our findings and explore their clinical application value.

We believe that these additions address your suggestion and provide a clear direction for future research. Your input has been instrumental in enhancing the comprehensiveness and forward-thinking nature of our manuscript.

Thank you once again for your insightful recommendation. We truly appreciate your support and guidance.

**Reviewer #4:**

**1. Mention limitations in methods and discussion that the study relies on diagnosis based on the codes and not a standardized clinical/radiological definition.**

**Response:**

Thank you for your valuable feedback regarding the limitations of our study. We have taken your suggestion to explicitly mention the reliance on diagnosis codes rather than a standardized clinical or radiological definition in both the Methods and Discussion sections. Here are the modifications we have made:

Modifications in the "Methods" Section

Original Text:

"The study population consisted of adult patients diagnosed with acute pulmonary edema within the MIMIC-IV database."

Modified Text:

"The study population consisted of adult patients diagnosed with acute pulmonary edema within the MIMIC-IV database, identified using ICD codes J810 and 5184. It is important to note that these diagnoses were based on billing codes rather than a standardized clinical or radiological definition, which may introduce variability in the diagnosis of acute pulmonary edema among patients."

Modifications in the " Limitations " Section

Original Text:

"Our study has several limitations."

Modified Text:

"Our study has several limitations. One significant limitation is the reliance on ICD codes for the diagnosis of acute pulmonary edema, rather than a standardized clinical or radiological definition. This approach may lead to misclassification bias, as the coding is dependent on the clinical judgment and documentation practices of healthcare providers. Additionally, the use of billing codes could potentially affect the accuracy of our findings, as these codes may not always reflect the true clinical picture. Future studies should consider incorporating standardized diagnostic criteria or additional validation methods to enhance the accuracy of the diagnosis."

We believe these changes will enhance the clarity and transparency of our study's limitations. We appreciate your guidance and are confident that these revisions will strengthen our manuscript.

Thank you once again for your constructive feedback.

**2. There is no adjustment for comprehensive illness severity scores (e.g., SOFA, SAPS II), which may confound the AG-mortality association. Recommendation: If feasible, include an illness severity score in a sensitivity analysis. If not, acknowledge this limitation explicitly.**

**Response:**

Thank you for your valuable suggestion to adjust for comprehensive illness severity scores such as SOFA (Sepsis-related Organ Failure Assessment) and SAPS II (Simplified Acute Physiology Score II) in our analysis. We have now included these adjustments in our statistical models and found that the results have been strengthened, confirming the robustness of our findings.

We have incorporated the SOFA and SAPS II scores into our multivariate Cox regression models to more accurately assess the relationship between anion gap and 28-day all-cause mortality, while controlling for illness severity.

Modifications Made:

• We have updated our statistical models to include both the SOFA and SAPS II scores as covariates.

- These adjustments were made to control for potential confounding effects that these scores might have on the association between anion gap and mortality.

Results of the Adjustment:

• After including the SOFA and SAPS II scores in our models, the association between anion gap and mortality remained significant and was in fact strengthened. This indicates that the relationship between anion gap and mortality is robust and not merely a result of uncontrolled confounding by illness severity.

• The hazard ratio (HR) for anion gap in the fully adjusted model (Model 3) was 1.11 (95% CI: 1.07-1.15, *P*<0.001), which is slightly lower than the crude model but still statistically significant, demonstrating the independent predictive value of anion gap after controlling for illness severity.

Updated Text in the "Methods" Section:

"We adjusted for potential confounders including age, gender, race, comorbidities, and illness severity scores (SOFA and SAPS II) in our multivariate Cox regression models."

Updated Text in the "Results" Section:

"Multivariate analysis including the SOFA and SAPS II scores confirmed anion gap as an independent predictor of 28-day all-cause mortality (HR=1.11, 95%CI: 1.07-1.15, *P*<0.001)."

We believe these changes will provide a more thorough and transparent discussion of our study's methodology and results. We appreciate your guidance and are confident that these revisions will strengthen our manuscript.

Thank you once again for your constructive feedback.

**3. Discussion section: Discuss how AG might be pragmatically used in clinical practice.**

**Response:**

Thank you for suggesting that we discuss the practical use of anion gap (AG) in clinical practice. We have added a new paragraph to the discussion section to address this point. Specifically, we have highlighted how AG can be pragmatically used as a readily available marker to assess the severity of metabolic disturbances in patients with acute pulmonary edema. Given its association with mortality, monitoring AG levels at admission and during hospitalization can help clinicians identify patients at higher risk for adverse outcomes. This information can be particularly valuable in resource-limited settings where more complex scoring systems may not be readily available. By incorporating AG into routine clinical assessments, clinicians can make more informed decisions regarding patient management and resource allocation.

We have added the following paragraph to the end of the discussion section (before the Limitations paragraph):

“In clinical practice, the anion gap can be pragmatically used as a readily available marker to assess the severity of metabolic disturbances in patients with acute pulmonary edema. Given its association with mortality, monitoring anion gap levels at admission and during hospitalization can help clinicians identify patients at higher risk for adverse outcomes. This information can be particularly valuable in resource-limited settings where more complex scoring systems may not be readily available. By incorporating anion gap into routine clinical assessments, clinicians can make more informed decisions regarding patient management and resource allocation.”

We believe that this addition strengthens the discussion by providing a clear and practical application of our findings in a clinical setting. We appreciate your suggestion and believe it has improved the manuscript.

Thank you once again for your insightful comments and for taking the time to review our manuscript.

**4. Figures: Make sure Axis labels and legends are self explanatory**

**Response:**

Thank you for your constructive feedback on our manuscript. We have taken your suggestion to ensure that the axis labels and legends in our figures are self-explanatory very seriously and have made the following revisions:

Figure 1: Flowchart of Patient Inclusion

We have revised the flowchart to include a clear title: "Patient Selection Process for Acute Pulmonary Edema Study" and added descriptive labels to each step for better understanding. The flowchart now explicitly states the number of patients at each stage, from initial identification to the final analysis cohort.

Figure 2: RCS Curve for the Anion Gap

The graph now includes a title: "Non-linear Relationship Between Anion Gap and 28-Day Mortality". The axis labels have been updated to "Anion Gap (mmol/dL)" on the X-axis and "Hazard Ratio" on the Y-axis. The legend now explains the significance of the reference point at 12 mmol/dL and the statistical values, providing clarity on the overall and non-linearity P-values.

Figure 3: Kaplan-Meier Survival Analysis Curves

The chart has been updated with a title: "Survival Analysis by Anion Gap Quartiles". The X-axis is labeled as "Time (Days)" and the Y-axis as "Survival Probability (%)". The legend now includes a brief explanation of the Kaplan-Meier method and the statistical significance indicated by the P-value, along with a clear presentation of the number at risk at each time point.

Figure 4: Forest Plot for Subgroup Analysis

The plot now features a title: "Subgroup Analysis of Anion Gap Impact on 28-Day Mortality". The axis labels are "Hazard Ratio" on the X-axis and "Subgroups" on the Y-axis. The legend provides a concise explanation of the hazard ratios and their 95% confidence intervals, along with the interaction P-values for each subgroup, enhancing the interpretability of the data presented.

We believe these revisions will significantly enhance the clarity and self-explanatory nature of our figures, making them more accessible to readers. We are grateful for your guidance and hope these changes meet your expectations.
