# Supplementary material for: Relationship between anion gap and 28-day all-cause mortality in patients with acute pulmonary edema: A retrospective analysis of the MIMIC-IV database
Source: PLoS One. 2025 Sep 26;20(9):e0333293. doi: 10.1371/journal.pone.0333293 (PMC12468886; doi:10.1371/journal.pone.0333293)
Supplement: Attachment — Submitted filename: Response_to_Reviewers_auresp_3.docx [file pone.0333293.s005.docx]

**Journal Requirements:**

**1. If the reviewer comments include a recommendation to cite specific previously published works, please review and evaluate these publications to determine whether they are relevant and should be cited. There is no requirement to cite these works unless the editor has indicated otherwise.**

**Response:**

Thank you for your valuable feedback. We have carefully reviewed the comments provided by the reviewers and would like to address the specific point regarding the citation of previously published works.

We understand the importance of citing relevant literature to support our findings and to situate our work within the existing body of knowledge. However, the reviewers did not provide specific recommendations to cite particular previously published works. Therefore, we have focused on ensuring that our references are comprehensive and relevant to the study's context and findings.

We have double-checked our references to confirm that they accurately reflect the current state of research in our field and are pertinent to our study's objectives, methods, and conclusions. We believe that our current list of citations adequately covers the necessary literature and provides a solid foundation for our work.

We appreciate the opportunity to clarify this matter and are confident that our manuscript is now fully aligned with the expectations for academic rigor and scholarly contribution.

Thank you for your continued guidance and support.

**2. Please review your reference list to ensure that it is complete and correct. If you have cited papers that have been retracted, please include the rationale for doing so in the manuscript text, or remove these references and replace them with relevant current references. Any changes to the reference list should be mentioned in the rebuttal letter that accompanies your revised manuscript. If you need to cite a retracted article, indicate the article’s retracted status in the References list and also include a citation and full reference for the retraction notice.**

**Response:**

Thank you for your attention to detail and for highlighting the importance of maintaining an accurate and up-to-date reference list in our manuscript. We have conducted a thorough review of our references to ensure completeness and correctness.

We have checked each citation against the most recent databases and have verified that none of the papers we have cited have been retracted. We understand the significance of retractions in the academic record and have taken the necessary steps to ensure that our references reflect the current state of the literature.

In the event that a retracted paper is deemed essential for the context of our study, we would follow the guidelines you provided. We would include a rationale for citing the retracted work within the manuscript text, clearly indicating its retracted status in the References list. Additionally, we would provide a citation and full reference for the retraction notice to maintain transparency and to acknowledge the current status of the cited work.

We have made no changes to the reference list in this revision, but we will adhere to these practices should the need arise in the future. We believe that our reference list is now complete and correct, and it supports the scholarly integrity of our manuscript.

We appreciate your guidance and are committed to upholding the highest standards of academic research and publication ethics.

Thank you for your continued support.

**Reviewer #4:**

**Q1. In grouping by anion gap: Quartile cut-offs appear to be arbitrary. A brief rationale or reference for these ranges should be added.**

**In results: Consider summarizing key differences between anion gap quartiles in text rather than listing every lab values.**

**Include a sentence comparing anion gap with other standard prognostic markers (e.g., lactate, BNP, or SOFA) to contextualize incremental value.**

We sincerely thank the reviewers for their constructive suggestions that helped us improve both the clarity and scientific rigor of our manuscript. Below are the point-by-point responses.

**1. Comment: “Quartile cut-offs appear to be arbitrary. A brief rationale or reference for these ranges should be added.”**

**Response:** Thank you for this helpful remark. In the revised Discussion we have added:

“The chosen cut-offs (< 10, 10–12, 12–15, > 15 mmol/L) mirror the ‘normal’, ‘borderline-high’, ‘high’ and ‘very-high’ strata repeatedly used in recent MIMIC-IV studies (Huang Y, Ao T, Zhen P, Hu M. Association between serum anion gap and 28-day mortality in critically ill patients with infective endocarditis: a retrospective cohort study from MIMIC IV database. BMC Cardiovasc Disord. 2024;24(1):585; Chen X, Yang Q, Gao L, Chen W, Gao X, Li Y, et al. Association Between Serum Anion Gap and Mortality in Critically Ill Patients with COPD in ICU: Data from the MIMIC IV Database. Int J Chron Obstruct Pulmon Dis. 2024;19:579-587).”

We appreciate your guidance in strengthening the justification of our grouping strategy.

**2. Comment: “Consider summarising key differences between anion-gap quartiles in text rather than listing every lab value.”**

**Response:** Thank you for this excellent suggestion. Instead of the previous long tabular recitation, the revised Results now reads:

“Across quartiles, higher anion-gap groups exhibited incrementally greater renal injury (median creatinine 0.8 → 1.4 mg/dL; median BUN 14 → 26 mg/dL) and lower haemoglobin (10.3 → 9.6 g/dL), while other laboratory parameters showed modest or non-systematic changes.”

We believe this condensed summary is much more reader-friendly while preserving the essential information.

**3. Comment: “Include a sentence comparing anion gap with other standard prognostic markers (e.g., lactate, BNP, or SOFA) to contextualise incremental value.”**

**Response:** Thank you for highlighting this important issue. The following sentence has been inserted in the Discussion:

“Although sofa, sapsii, lactate and BNP are established predictors, admission anion gap remained independently associated with 28-day mortality after adjustment for these variables, suggesting that it conveys additional prognostic information beyond that provided by conventional scores or lactate levels.”

Once again, we are grateful to the reviewers for their insightful feedback, which has substantially improved the quality of our manuscript.
